# A method of characterising the complex anatomy of vascular occlusions and 3D printing biomimetic analogues

**DOI:** 10.1111/joa.13648

**Published:** 2022-03-07

**Authors:** Muireann O'Reilly, Rachel Beatty, Shauna McBride, Benjamin Brennan, Peter Dockery, Garry P. Duffy

**Affiliations:** ^1^ Discipline of Anatomy and Regenerative Medicine Institute, School of Medicine, College of Medicine, Nursing and Health Sciences National University of Ireland Galway Galway Ireland; ^2^ CÚRAM, SFI Research Centre for Medical Devices National University of Ireland Galway Galway Ireland; ^3^ SFI Research Centre for Advanced Materials and Bioengineering Research (AMBER) Trinity College Dublin & National University of Ireland Galway Galway Ireland

**Keywords:** biomimetic, chronic total occlusion, medical device testing, microcomputed tomography, soft tissue imaging, vascular occlusion

## Abstract

Chronic total occlusions (CTOs) occur in approximately 40% of individuals with symptomatic peripheral arterial disease and are indicative of critical limb ischaemia. Currently, few medical devices can effectively treat CTOs long‐term, with amputation often required. This is due to a lack of knowledge of CTO anatomy, making device design and testing difficult. This study is a proof‐of‐concept study, which aimed to develop a workflow for further characterising the complex multi‐material anatomy of CTOs and creating 3D models of CTO components, which may be useful in producing a vascular CTO biomimetic for device testing. Here, we establish such a workflow using samples of atheromatous plaques. We focus on a high‐resolution, non‐destructive microcomputed tomography (μCT) technique which enables visualisation of occlusion anatomy at a greater resolution than computed tomography angiography (CTA), which is the typical modality used for CTO clinical visualisation. Four arteries (*n* = 2 superficial femoral; *n* = 2 popliteal) with evidence of atheromatous plaques were cut into 8 cm segments, which were then stained with iodine and scanned at low resolution, with calcified regions rescanned at high resolution. Resulting files were manually segmented to generate 3D models, which were then 3D printed in resin using a stereolithography printer to produce parts suitable for creating a biomimetic. In total, μCT files from three arterial segments (*n* = 2 high resolution, *n* = 1 low resolution) were deemed suitably calcified for segmentation, and thus were segmented to produce 3D models. 3D models of the arterial wall, intima and atheromatous calcium deposits from a high‐resolution popliteal artery scan were successfully 3D printed at several scales. While this research is at an early stage, it holds great promise. The workflow for segmentation and 3D printing various components of an atheromatous plaque established here is replicable and uses software and equipment which are accessible to research laboratories in both academia and industry. The ability to print detailed models on a desktop 3D printer is unprecedented and can be improved further, which is promising for future development of biomimetics with multi‐material detail of both soft tissue and calcified components of a vascular occlusion. Indeed, this workflow provides a solid foundation for future studies of CTO anatomy and the creation of true, multi‐material CTO biomimetics. Such biomimetics may enable the development of improved interventional devices, as they would mimic the general in vivo CTO environment. As this method cannot be applied in vivo*,* we cannot yet produce patient‐specific biomimetics, however, these analogues would still be important in device development, which would improve patient outcomes in critical limb ischaemia.

## Graphical abstract

We have described a microcomputed tomography method which provides multi‐material detail on the calcified and soft tissue components of vascular occlusions. This is enables models of various components of the occlusion to be 3D printed, for use in the creation of biomimetic analogues. Having this detail on occlusion anatomy is important for the development and testing of interventional medical devices, particularly for treatment of chronic total occlusions.

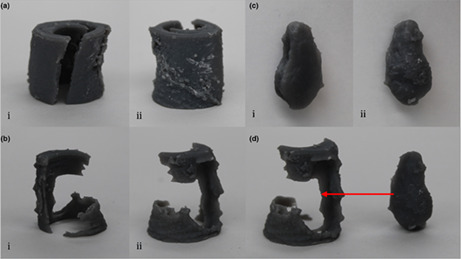

## 1 INTRODUCTION

Peripheral arterial disease (PAD) is the narrowing or occlusion of the arteries outside of the heart and brain. In 2019, the global prevalence of PAD was 113 million, with 74,100 associated deaths (Roth et al., 2020). PAD affects 18% of the population between 60 and 90 years (Hamur et al., 2017). Chronic limb ischaemia (CLI) is a form of PAD affecting the lower limbs, the most advanced state of which is critical limb ischaemia. The definition of critical limb ischaemia is not standardised, making epidemiological studies difficult, however, it has a prevalence of approximately 1.3% in the USA (Duff et al., 2019; Fereydooni et al., 2020). CLI prevalence continues to rise as cases of comorbidities rise. These include diabetes, obesity, high systolic blood pressure and tobacco use (Roth et al., 2020). A quarter of patients with critical limb ischaemia die within 1 year of diagnosis, while 45% of those who require an amputation die within a year of the procedure. Earlier diagnosis of CLI is associated with better patient outcomes, as lifestyle changes such as increased activity and dietary improvements can be implemented. Severe cases necessitate surgical intervention (Hamur et al., 2017).

A chronic total occlusion (CTO) is a complete arterial occlusion, which entirely prevents blood flow distal to it, present for 3 months or more. CTOs occur in up to 40% of patients with symptomatic PAD (Hamur et al., 2017). The Trans‐Atlantic Inter‐Society Consensus Document on Management of Peripheral Arterial Disease (TASC II) classifies CTOs as the common femoral artery (CFA) or superficial femoral artery (SFA) (>20 cm, involving the popliteal artery) or popliteal artery and proximal trifurcation vessels as Type D lesions, the most severe type. Type D lesions are typically heavily calcified and indicative of critical limb ischaemia (Norgren et al., 2007).

CTOs are caused by atherosclerosis, which is the build‐up of plaque in the intima of the arterial wall, along with endothelial dysfunction, lipid accumulation and inflammation. This leads to eventual partial or complete arterial occlusion (Norgren et al., 2007). With CTOs, a necrotic lipid core forms and is capped by the addition of fibrous tissue. This fibrous plaque can now form a dominant lesion and completely occlude the vessel. Calcification of the necrotic core occurs gradually, hardening the plaque and making the vessel increasingly stiff (Insull Jr, 2009). A study by Herisson et al. (2011) concluded that 93% of femoral plaques studied were fibrocalcific (according to a plaque classification system described in Dalager et al., 2007), and demonstrated sheetlike calcification, nodular calcification or osteoid metaplasia.

In recent years, studies have characterised advancements in endovascular treatments which have made it possible to cross CTOs with a guidewire and specialised catheters. Kondapalli et al. (2018) examined patient outcomes with intraluminal and subintimal vessel crossing strategies, concluding that while intraluminal techniques were used more frequently in their sample, subintimal techniques (creating a neolumen between the intima and media or adventitia) are preferred for heavily calcified, long lesions or where an intraluminal technique has failed. However, re‐entry of the guidewire into the true lumen is challenging, with intravascular ultrasound and a re‐entry catheter often required. Furthermore, Saab et al. (2018) examined the antegrade and retrograde approach strategies for these crossing techniques and devised a new CTO classification system using plaque cap morphology (CTOP) to determine the optimal direction from which to cross a CTO. Despite the ability to achieve endovascular access, there are difficulties. A major issue for CTO treatment is that endovascular crossing alone is not sufficient to rectify CTOs long‐term, as calcification makes device deployment and maintenance of vessel patency difficult. Most balloons are not sufficiently strong to push calcified plaques aside (Sheeran & Wilkins, 2018), while restenosis rates with stenting are up to 21% (Abbott & Williams, 2007).

Given the varying multi‐material nature of lower extremity CTOs, further understanding their complex anatomy and exact composition is critical in advancing treatment options. This knowledge can also be used to develop biomimetic analogues of CTOs which may prove valuable in improving interventional tools. We recognised that true CTO samples may be difficult to obtain, given the limited cadaver sample available for analysis. Therefore, the following is a proof‐of‐concept study, which establishes a workflow for analysing the multi‐material anatomy of vascular occlusions (in this case, we analyse atheromatous plaques). Atheromatous plaques are a precursor to CTOs; thus, these models are useful for examining the location and structure of plaques which have not yet become clinically problematic. Furthermore, if these plaques (which are considerably smaller than CTOs and are localised within the arterial wall, rather than the lumen) can be imaged and reproduced at high quality, it is likely the same could be done for true CTO samples in future. This is important given the clinical relevance of CTOs.

Therefore, the primary objective of this study was to demonstrate a novel workflow for imaging CTO samples and using the anatomical detail obtained to create useful CTO biomimetics. This workflow uses microcomputed tomography (μCT) scans of cadaveric arterial samples to investigate plaque anatomy, by visualising both calcified and soft tissue components, segmenting them and printing the resulting files as three‐dimensional (3D) models. When applied to a CTO sample, the 3D models could then be used to better characterise the composition of CTOs and potentially to mould and assemble a multi‐material CTO biomimetic for testing interventional tools. As the software and equipment used are common to research laboratories worldwide, the optimised workflow can be replicated easily and affordably, to create models for use in device testing or potentially for planning surgical interventions.

## 2 METHODS

### 2.1 Isolation of arteries

Arteries were obtained from the lower limbs of two cadavers (77‐year‐old female, right limb; 84‐year‐old male, left limb) which were embalmed in traditional embalming fluid (42.3% water, 21.1% methanol, 21.1% glycerine, 7% phenol and 8.5% formaldehyde). Cadavers were selected based on availability, as there was a limited population size available for this study, while embalming was performed under gravity. The study protocol was approved by the NUI Galway Research Ethics Committee. Cadavers were bequeathed to the School of Medicine at NUI Galway for further advancement of medical knowledge. This is covered by legislation governing the practice of Anatomy in the Republic of Ireland (Medical Practitioners Act 2007). The arteries isolated were the SFA (from the point of branching of the profunda femoris artery down to its point of entry into the adductor hiatus) and the popliteal artery (from the adductor hiatus down to its trifurcation in the leg into the anterior tibial, posterior tibial and peroneal arteries). After dissection, arteries were stored in 70% ethanol.

### 2.2 Assessment of calcification

Arteries were longitudinally incised and examined for evidence of calcified atheromatous plaques. Visual evidence included white plaques, separation of the intima from the media and protrusions/unevenness in the arterial wall. The wall was also palpated and examined for any unusual thickness or brittleness. Arteries with evidence of disease were cut into 8 cm segments and resubmerged in 70% ethanol.

### 2.3 Sample staining and μCT scanning

A non‐destructive, high‐resolution μCT technique for imaging whole arterial samples as previously described in Robinson et al. (2021) was used.

In brief, samples were stained in a 1% w/v iodine: 100% ethanol solution for 24 h. Samples were then washed in two changes of 100% ethanol for 5 min each to remove excess iodine. Samples were then placed in μCT sample holders, positioned using polyethylene packing foam and completely covered in 100% ethanol for imaging. Holders were capped and sealed with parafilm. μCT images were taken using a Scanco μCT 100 scanner (Scanco Medical microCT 100 system, https://www.scanco.ch/microct100.html, RRID:SCR_017119, Scanco Medical). Firstly, low‐resolution scans (7e+0.1 kV with slice thickness of 34 μm and pixel size of 34 μm) were taken of each whole 8 cm segment to assess the level of calcification present. Heavily calcified samples were rescanned at high‐resolution (7e+0.1 kV with slice thickness of 11 μm and pixel size of 11 or 57 μm) at the calcified subsection of the segment.

### 2.4 Assessment of detail on μCT scans

Resulting μCT files were opened in Dragonfly (https://www.theobjects.com/dragonfly/index.html, Object Research Systems) to determine the level of detail achieved on calcification. Files with obvious calcification visible were imported and opened in Mimics (http://biomedical.materialise.com/mimics, RRID:SCR_012153, Materialise NV).

### 2.5 Segmentation in mimics

Once files were opened in Mimics, contrast was set to the bone contrast setting to make calcium appear bright white. Segmentation was performed mainly in the axial view. The same process was applied to low‐ and high‐resolution scans. Masks were applied to segment calcium deposits, the arterial wall (the adventitial, medial and intimal layers were also segmented individually for some samples), the arterial lumen and fatty deposits. In all scans, contrast significantly differed between soft tissues (e.g., the arterial wall) and calcium deposits, allowing thresholding to be used to segment these components. Region growing functions were used where there was a considerable amount of scatter in the thresholded mask, which would make 3D printing difficult. Manual segmentation was used for masking fatty deposits and individual arterial wall layers. Videos were captured in Mimics using PowerPoint (https://www.microsoft.com/en‐us/microsoft‐365/powerpoint, Microsoft Corporation). Table 1 contains details of the segmentation procedure.

**TABLE 1:** Detailed procedure for segmenting μCT files of diseased cadaveric arterial samples in mimics

Mask number	Colour	Description	Operation for creation
1	Green	Calcium deposits	Thresholding
2	Yellow	Everything except calcium deposits	Thresholding
3	**Cyan**	**All calcium deposits (no scatter)**	Boolean Operation: Mask 1 – Mask 2
4	**Orange**	**Major calcium deposits**	Region Growing on Mask 3
5	Fuchsia	Arterial wall	Thresholding
6	Red	Black areas (background and arterial lumen)	Thresholding
7	Blue	Arterial wall (some scatter)	Boolean Operation: Mask 5 – Mask 6
8	**Magenta**	**Arterial wall (no scatter)**	Region Growing on Mask 7
9	Yellow‐Green	Arterial lumen	Mask applied to threshold black areas, manually segmented to remove background and copied 10 slices. Re‐segmented every 30 slices or as necessary, applied every 50 slices
10	**Purple**	**Arterial lumen (no scatter)**	Region Growing on Mask 9
11	Pale Green	Fatty deposits	Mask applied to threshold entire field of view, cleared and manually segmented every 10 slices to add fatty deposits, interpolated and applied every 50 slices
12	**Green**	**Fatty deposits (no scatter)**	Boolean Operation: Mask 11 – Mask 3
13	**Turquoise**	**Arterial wall (with space for fatty deposits)**	Boolean Operation: Mask 8 – Mask 12
14–17	**Lilac** **Pink** **Light blue** **Peach**	**Arterial wall layers**	Duplicate Mask 8 and manually remove the intima = Mask 14 Boolean Operation: Mask 8 – Mask 14 = **Intima** (Mask 15) Duplicate Mask 14 and remove media = **Adventitia** (Mask 16) Boolean Operation: Mask 14 – Mask 16 = **Media** (Mask 17)

*Note*: 3D objects were calculated in Mimics for masks highlighted in bold. These could be exported as STL files and 3D‐printed.

### 2.6 3D printing

STL files were opened in PreForm (https://formlabs.com/eu/software/, Formlabs). The files were automatically oriented and support rafts were autogenerated. Models were then reoriented if necessary and rafts edited to ensure adequate support was provided for successful printing. A Form 3L stereolithography (SLA) printer (https://formlabs.com/eu/3d‐printers/form‐3l/, Formlabs) was used for 3D printing. Models were printed in grey resin at 1X scale (the original scale of the model as exported from Mimics). The files were printed at 1.5X, 2X, 3X, 4X and 5X scale also (X times the original scale in all directions). Once models were printed, they were removed from the printer bed and washed in 100% isopropanol in a Formlabs Form Wash station (https://formlabs.com/eu/post‐processing/wash‐cure/, Formlabs) for 15 min to remove any excess resin. Once washed, the models were UV cured in a Formlabs Form Cure station (https://formlabs.com/eu/post‐processing/wash‐cure/, Formlabs) at 60°C for 30 min.

## 3 RESULTS

### 3.1 Analysis of cadaveric arterial samples

**FIGURE 1:**
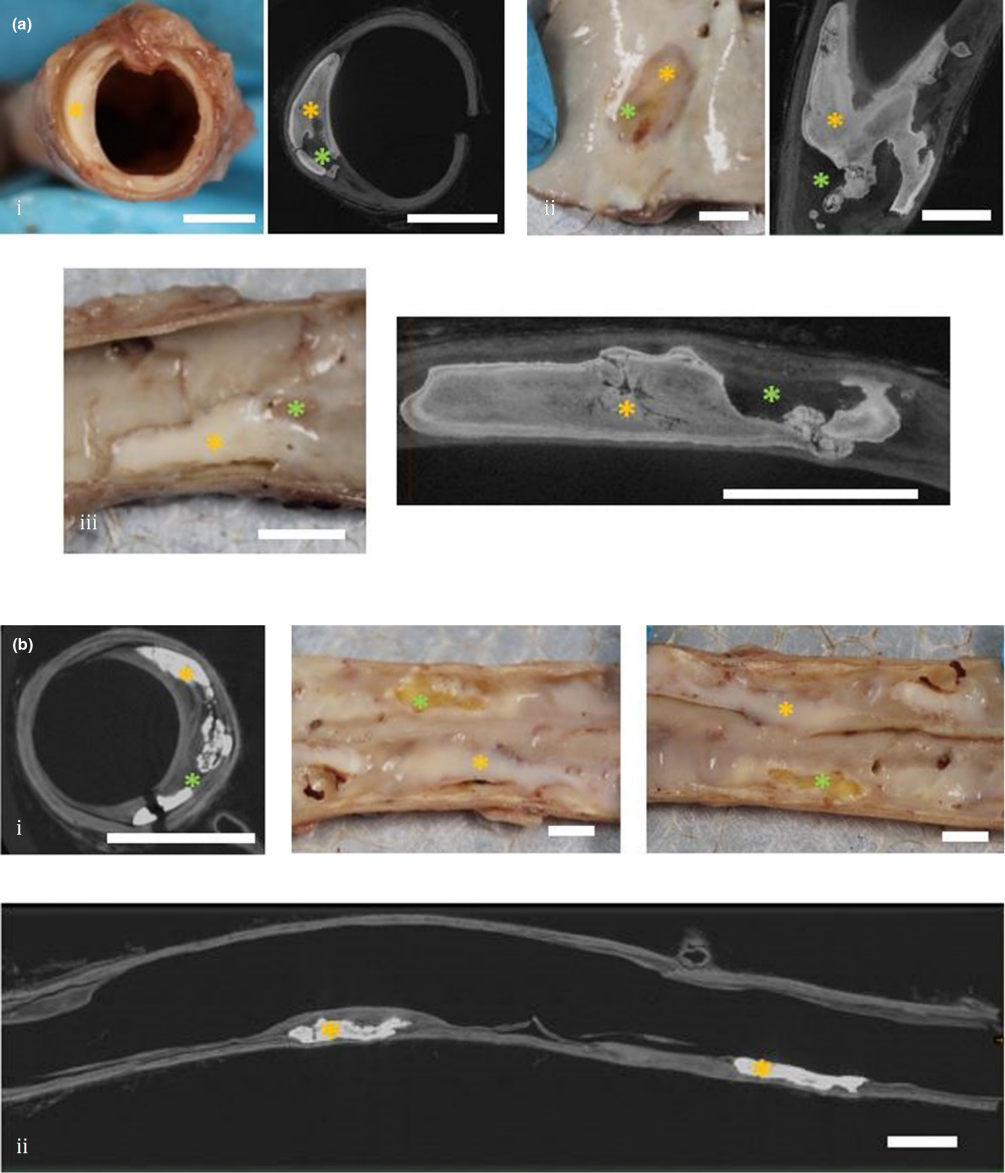
Overview of diseased cadaveric arterial samples and corresponding μCT scans. Gross anatomical samples of diseased cadaveric arteries are shown along with corresponding microcomputed tomography (μCT) scans. The corresponding features on each are marked accordingly. (a) High‐resolution scan of a segment of SFA. Note that in high‐resolution scans only the calcified region of the arterial segment is scanned. i: Axial view, ii: Sagittal view, iii: Coronal view. (b) Low‐resolution scan of a segment of popliteal artery. i: Axial view, ii: Coronal view ‐ note how in low‐resolution scans the entire length of the arterial sample is scanned. Cadaveric samples in B are shown in the coronal view. All samples photographed are from the right lower limb of a 77‐year‐old female cadaver. Key: Orange asterisk: calcium deposits, Green asterisk: fatty deposits (colours correspond to 3D models in Figure 2). Scale bars: 5 mm

The preliminary assessment of the four arteries showed that each had evidence of calcification and/or fatty deposits (see gross samples in Figure 1) in the inner layers of the arterial wall, particularly in the intima, which was visible when the interior of the artery was examined. These lesions, termed atheromatous plaques, are examples of endothelial dysfunction – while not true CTO samples, we deemed the dysfunction significant enough to be used to demonstrate our workflow and thus imaged them in the μCT scanner.

### 3.2 Assessment of detail on high‐resolution μCT scans

A high level of detail could be obtained from both low‐ and high‐resolution scans when viewed in Dragonfly. Both provided excellent resolution compared to 2 mm axial CTA scans (CTA is the primary imaging technique for CTO diagnosis and interventions according to Courtney et al., 2008, hence it was used for comparison here). The iodine staining created high contrast which aided in distinguishing calcification and associated soft tissue structures such as fatty deposits (which appear black) and the arterial wall (which appears grey) (Figure 1).

### 3.3 Segmentation in mimics

**FIGURE 2:**
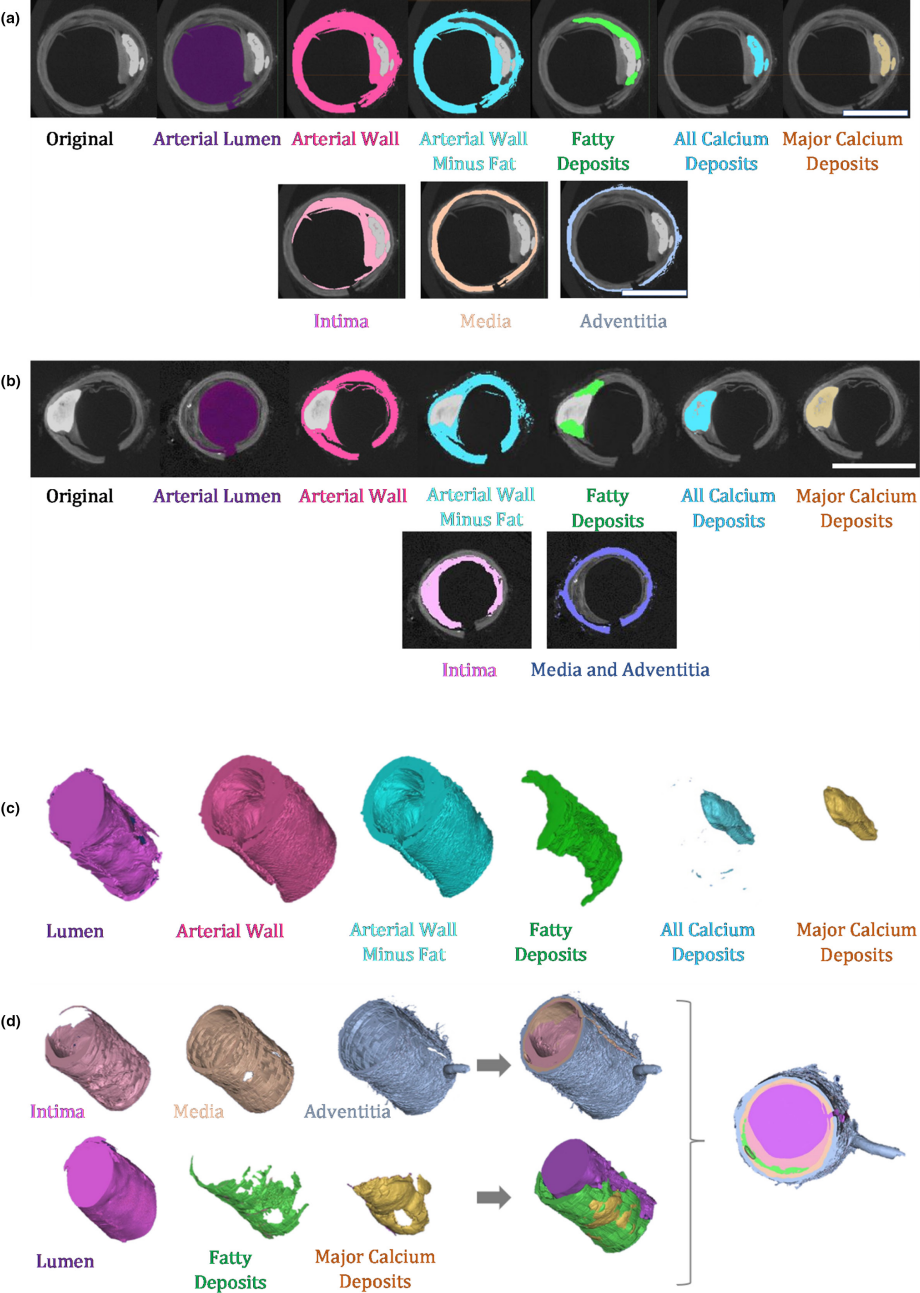
Segmentation of μCT scans of cadaveric arterial samples in Mimics. (a) Axial view of a low‐resolution scan of a segment of popliteal artery with several masks applied, demonstrating the level of detail which can be obtained with microcomputed tomography (μCT) scans and the segmentation possibilities. (b) Axial view of a high‐resolution scan of another segment of the same artery with the same masks applied. (c) Examples of the basic 3D models which can be generated from the μCT scans in Mimics. Models were generated from the high‐resolution scan in (b) (see masks in first row). (d) Advanced models which can be generated in Mimics. Models were generated from a subsection of the low‐resolution scan in (a) (see masks in second row). Models were generated from μCT scans of arterial samples taken from the right lower limb of a 77‐year‐old female cadaver. Scale bars: 5 mm, the same scale bars apply to the corresponding 3D models (a and d, b and c)

Three μCT files were segmented in Mimics. All were scans of arterial segments from the female cadaver with evidence of arterial atheroma – two were popliteal artery scans (*n* = 1 low‐resolution, *n* = 1 high‐resolution) and one was a high‐resolution SFA scan. No scans of samples from the male cadaver were suitably calcified for segmentation. Masks 1–13 in Table 1 were applied to all scans and corresponding 3D objects were generated (Figure 2a–C).

**FIGURE 3:**
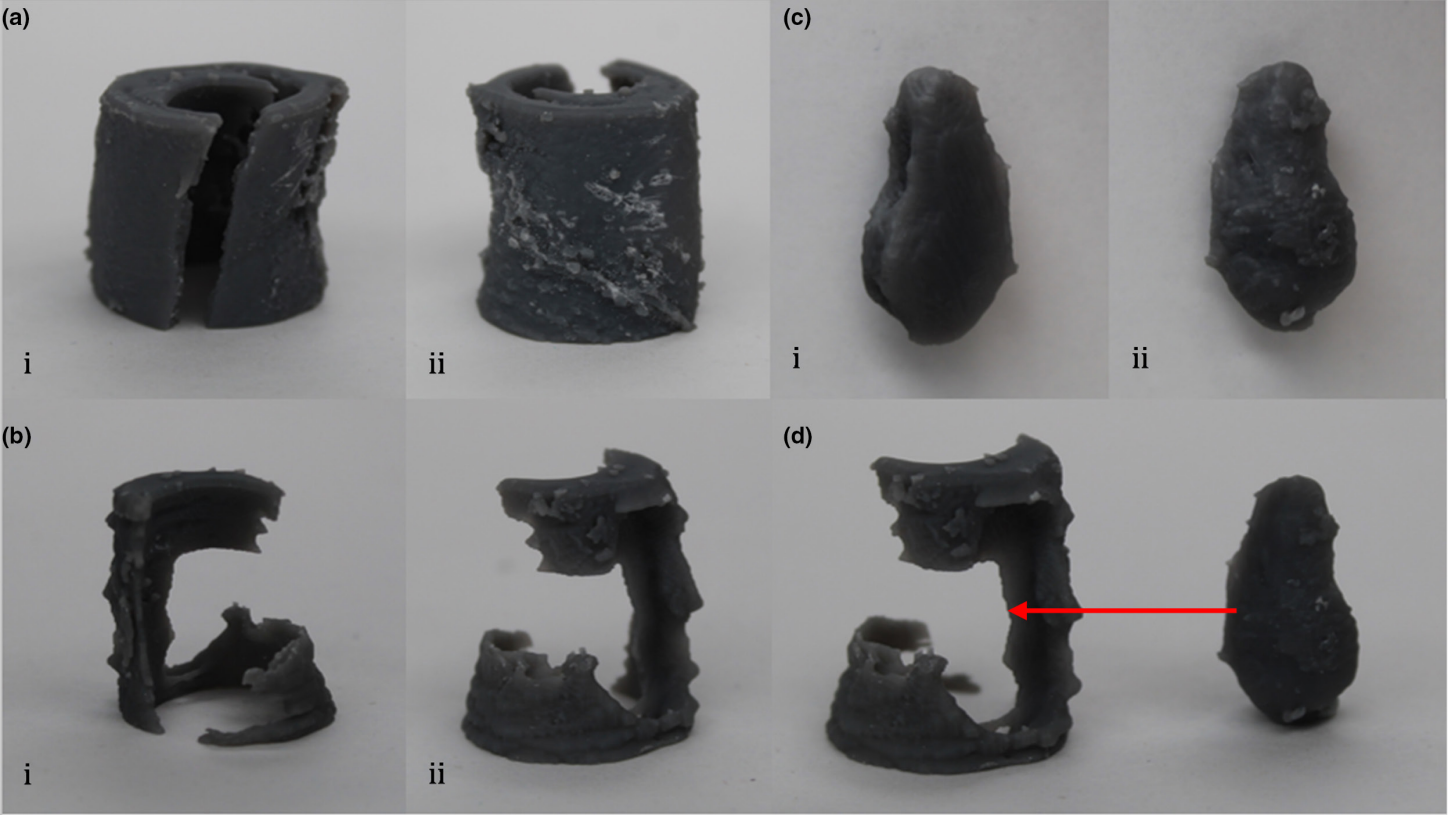
Overview of 3D‐printed models. All models were printed in grey resin on a Formlabs Form 3L 3D printer and were created from a high‐resolution microcomputed tomography (μCT) scan of a popliteal artery sample taken from the right lower limb of a 77‐year‐old female cadaver. The model shown is a 3X print (the scale of the STL file was increased to produce models three times the size of the original in all directions), which is close to the actual size of the popliteal artery. (a) Arterial wall i: Anterior view, ii: Posterior view. (b) Intima i: Anterior view, ii: Posterior view. (c) Atheromatous calcium deposits i: Anterior view, ii: Posterior view. (d) Posterior view of the arterial intima and calcium deposit – the red arrow shows where the calcium deposit is located within the intima

Both popliteal artery files provided a high level of detail on the structure of the arterial wall, enabling some or all constituent layers to be individually segmented (Masks 14–17 in Table 1; Figure 2a,b) and 3D objects to be calculated (Figure 2d).

The high resolution of the μCT scans produced smoother and more detailed 3D models compared to 2 mm axial CTA scans. Despite the difference in resolution between low‐ and high‐resolution μCT scans, there was only a slight difference in the quality of the 3D objects produced from each. Ultimately, for this application low‐resolution scans provide plentiful detail, as is evident in Figure 2d. Due to the larger area scanned in low‐resolution scans, the resulting 3D objects were longer than those produced from high‐resolution scans (see Video S1). However, it was possible to segment only at the calcified subsection (as shown in Figure 2d). Essentially, rather than segmenting the entire low‐resolution file (which covers an entire 8 cm arterial segment), the range of slices within which an atheromatous plaque is visible are identified and the segmentation is performed only within this range. 3D models are still generated as normal, but rather than covering the entire slice range of the scan, the model covers only the range of interest. This saves significant time during both scanning and segmentation, while still providing all necessary detail to 3D print biomimetic analogues. Video S1 gives an overview of all scans and corresponding 3D objects.

### 3.4 3D printing

The 3D models generated from the high‐resolution scan of the popliteal artery in Mimics were exported as STL files and printed successfully at 1X, 1.5X, 2X, 3X, 4X and 5X scale. The scale here refers to the setting used for printing in the PreForm software – at 1X scale, the model was printed using the original scale of the STL, at 2X scale, the scale setting in PreForm was increased to 2, thus the model was printed at 2 times the size of the original in all directions. The models printed were – the arterial wall (Mask 8 in Table 1; Figure 3a), the intima (for this μCT file only the intima could be individually segmented from the arterial wall – see Mask 15 in Table 1; Figure 3b) and the major calcium deposits (smaller “scattered” pieces of calcium were removed in Mimics as they were too small to print successfully – see Mask 4 in Table 1; Figure 3c).

The 1X and 1.5X models were smaller than the original cadaveric sample, and due to the delicate structure of the arterial wall and intima models, these broke upon removal of the support rafts. Calcium models printed at this scale remained intact. The 2X, 3X, 4X and 5X models were successfully removed from support rafts without breakage – of these, the 2X and 3X were closest to the true size of the popliteal artery. Despite the use of resin, the surface topography was quite detailed, although the sanding required to smooth the surface after removal of the support rafts did affect this. The larger models were robust, while printing the intima individually allowed the exact location of the calcium deposit within the arterial wall to be identified (see Figure 3d) and could be useful for constructing soft tissue structures around when creating a multi‐material biomimetic.

## 4 DISCUSSION

### 4.1 Importance of knowledge of CTO anatomy and biomimetic creation

The main aim of this study was to describe a clinically relevant application for a previously described, high‐resolution μCT technique by imaging diseased cadaveric arterial samples, demonstrating how both calcified and soft tissue components of a vascular occlusion can be visualised, and how this might be applied to create realistic biomimetics to test medical devices. To the best of our knowledge, this concept is novel and no papers have been published describing a similarly replicable method of imaging and 3D printing diseased arterial samples to create a useful biomimetic. While we could not obtain true CTO samples for analysis in this study, the ability to obtain detail on the multi‐material components of an atheromatous plaque suggests this method could be used to visualise CTO samples in future. Indeed, this workflow is imperative for future studies of CTO anatomy, as it provides the foundations for successfully visualising their various components and 3D printing models of these. This is a promising development – currently, there are few devices that can successfully treat a CTO long term. The lack of effective interventional tools is largely due to a lack of knowledge of the complex geometric anatomy of CTOs, which stems from both inadequacies in imaging techniques and CTO severity – amputation is often the most effective option for preventing unwanted cardiovascular events short‐term (Kinlay, 2016). Therefore, there is little opportunity for studying CTO composition in vivo. Studies have shown that human‐like peripheral CTOs can be induced in pigs by eliciting an inflammatory response with polymer‐coated stents, however, these are not spontaneous and the process for their induction is costly and time‐consuming (Kim et al., 2015). The models created here use a simple workflow, with widely available and affordable materials. This makes it accessible to research laboratories, both in academia and industry, which is important for improvement of interventions. Previous studies have characterised optimal endovascular access routes (Kondapalli et al., 2018; Saab et al., 2018). Therefore, having a workflow for creating a realistic biomimetic which is accessible to individuals with a variety of expertise can build on this to aid device innovation and improve patient outcomes in the long‐term.

Despite the promise this workflow holds, we do acknowledge that there are some limitations to the present study, given that it is intended as proof‐of‐concept. As previously mentioned, none of the samples used were true CTO samples – rather, they were samples of atheromatous plaques. However, we have argued that they are sufficient for demonstrating this workflow, and we believe it can be applied to CTO samples successfully in future. As this process is performed ex vivo, ideally amputated limbs from critical limb ischaemia patients would be donated from local hospitals and the occluded artery isolated, with this high‐resolution μCT technique used to image the CTO. This is currently being developed at NUI Galway as a promising way forward for increasing knowledge of CTO composition and would enable the development of more realistic biomimetics.

Secondly, the STL files 3D‐printed here were of the high‐resolution popliteal artery scan, in which only the intima could be individually segmented from the arterial wall due to the significant disruption caused by the calcification which was present. However, we do not believe that this affects the validity of the workflow. Indeed, we are currently exploring a method of creating biomimetics which does not require the arterial wall to be 3D‐printed. Instead, it involves 3D printing models of the arterial lumen and calcium deposits. We are particularly interested in the latter, as they are typically the most problematic component of a CTO to cross, given that they occlude the entire arterial lumen. We aim to mould the 3D‐printed arterial lumen model in silicone, to create a silicone mould of the artery itself, with the same inner diameter as the actual vessel. Ideally, this silicone would be transparent, to allow device interactions with calcium deposits to be visualised, but also have adequate durability to ensure that the 3D‐printed lumen piece could be removed from within without damaging the mould. Once the silicone mould is cured, the 3D‐printed calcium deposits can be placed within the lumen in the correct geometric positions. Furthermore, as soft tissue detail (particularly fatty detail) can be obtained from these high‐resolution scans following iodine staining, this could be simulated in a realistic manner to create an effective biomimetic. In the future, we plan to use gelatin solutions of various viscosities to simulate the soft tissue components of the CTO – ultimately, these components pose no difficulty to the passage or deployment of a device, making a soft material like gelatin an effective mimic.

Indeed, histological studies of the CTO could be conducted to further characterise the soft tissue components – for example, with appropriate staining greater anatomical detail may be obtained on the fibrous tissue caps. For future studies, it would also be interesting to print a low‐resolution file with each arterial wall layer individually segmented to determine the quality and compare it to that of the models created here.

### 4.2 Application of μCT scanning technique

This study builds upon a previously published paper by Robinson et al. (2021) and demonstrates a specific use for the high‐resolution μCT scanning technique described in that paper. The μCT technique proved very effective for this application – iodine staining provides excellent contrast between calcium deposits and soft tissue, such as fat. This provides greater detail on the composition and structure of atherosclerotic plaques. Furthermore, the ability to segment the individual layers of the arterial wall was promising – while this was achieved for the CFA previously (Robinson et al., 2021), this was the first study to demonstrate this on the popliteal artery, while the ability to do so from low‐resolution scans was unprecedented and may prevent the requirement for further time‐consuming high‐resolution scans.

### 4.3 3D printing

The workflow for segmentation established here was successfully applied to generate 3D‐printed models. These printed models appeared as they had in the Mimics software, thus the detail visible was retained during printing. Here, printing models to scale was achieved with a desktop SLA printer, which prints layer‐by‐layer in resin. This type of 3D printer would not typically be used for printing high‐resolution anatomical models ‐ objects cannot be printed directly onto the printer bed, so support rafts are generated, the removal of which can affect surface topography. This method worked well for this study, with models appearing as in Mimics and showing good topological and structural detail. There were scaling issues with the print, however, this was unsurprising, as this printer is typically used for printing models drawn in computer‐aided design packages, with known dimensions. STL files were directly exported from Mimics and were not opened in any other software prior to printing – further investigation is required to determine the exact cause of the size discrepancies (i.e., why the 1X print was not the size of the original sample), but this may stem from the high resolution at which the arteries were imaged – we aim to investigate this in future.

For future studies, a ceramic 3D printer, such as the Lithoz CeraFab Multi 2M30 (https://www.lithoz.com/en/our‐products/3D‐printers#CeraFab%20Multi, Lithoz GmbH) which uses LED light to polymerise a photosensitive, ceramic powder matrix into solid ceramics would be preferable. These printers do not require support rafts and can print high‐resolution, geometrically complex parts. This would preserve the topography of our samples and enable higher detail to be achieved on the models. Furthermore, the ceramic material would more closely mimic the properties of bone than resin – indeed, the mechanical properties of the different printing mediums available could be tested and compared to those of bone, which would enable the optimal material to be chosen for future applications. However, the ability to create these models from an SLA printer is promising, as these are relatively accessible to most research laboratories and thus may enable the widespread use of this technique. For CTO models, the calcium deposits are the most critical component, given they are the most difficult component to cross. Their solid structure means they can be easily printed on an SLA printer, which may be effective for preliminary studies.

### 4.4 Limitations

There are other limitations to this study, aside from those mentioned above, which can be rectified in our future work. Firstly, it would be beneficial to add additional validation to the thresholding and masking of structures in μCT files. This could be done by having multiple maskers, i.e., by having multiple individuals mask the same file to determine if there is any inter‐observer variability. We anticipate that there will be many files available for masking in future studies, with the increased sample size also useful in reducing any artefacts.

Furthermore, the sample size in this study was small, as we were limited by cadaver availability. This explains why only three μCT files were available for segmentation – ultimately, the male cadaver did not supply any samples which were suitably calcified to warrant further segmentation. With a larger sample, this cadaver could have been excluded from analysis entirely. However, given the proof‐of‐concept nature of this study, we do not see these as critical issues – ultimately, this workflow is being designed for application to fresh tissue samples from patients. The nature of the embalming process (by gravity) does influence arterial size – it is likely samples here are larger than they would be physiologically, due to fluid retention. The size increase is impossible to quantify, as it varies depending on the physical characteristics of the cadaver and the amount of embalming fluid required. While this is not ideal, we do not believe that it impacts on the validity of this workflow for application to CTO samples, given that they will be fresh samples.

### 4.5 Applications of CTO biomimetics

We believe that this workflow is an important foundation for the creation of effective CTO biomimetics, which will form a critical component of device testing. As mentioned in the introduction, there have been several studies into optimal crossing strategies for CTOs in recent years (Kondapalli et al., 2018; Saab et al., 2018). While these have shown promising results, it may be useful to perform further validation. While intraluminal crossing strategies are widely favoured by interventionalists, subintimal angioplasty and subsequent re‐entry to the true lumen are important in treating CTOs, given they have high success rates, particularly with longer lesions. A recent systematic review of subintimal angioplasty and re‐entry devices found that when subintimal angioplasty alone was employed, the success rate for revascularisation was 92.5%, while when used along with re‐entry devices, the success rate was 88.3%. An interesting finding was that complication rates tended to be higher when CLI was most prevalent, calcified lesions had a greater mean length, and the age of the individual was higher (Kokkinidis et al., 2020). This suggests that further studies of the efficacy of this technique may be useful, particularly studies involving heavily calcified, long CTOs from older individuals. If a biomimetic were to be created from such a sample, it could be reused several times for device testing and may be useful in determining the optimal crossing strategy for complex lesions.

Furthermore, even when crossing is achieved, there are a range of treatment options available, with none perfectly effective. Indeed, there is a paucity of devices which can maintain vessel patency long‐term, even if originally successful (Sheeran & Wilkins, 2018). The efficacy of endovascular techniques with lesions >15 cm (i.e., TASC II type C or D lesions) is debatable, with bypass typically preferred in these instances (Zeller et al., 2014). However, Zeller et al. (2014) conducted a study comparing the outcomes between drug‐coated balloon angioplasty and drug‐eluting stents where mean lesion length was 19 cm. They found that there was no significant difference between restenosis rates and target lesion revascularisation using these two strategies after 1 year, and the results were comparable to those seen in studies of shorter lesions (Zeller et al., 2014). This suggests that these devices hold promise, and may be improved with further innovation, making them effective for use in CTO treatment. Other clinical trials have examined the efficacy of nitinol self‐expanding stents, and their ability to restore and retain vessel patency. The STELLA (**STE**nting **L**ong de **L**'**A**rtère fémorale superficielle) trial evaluated these qualities in TASC II type C and D lesions, where the average length stented was 260 ± 180 mm. It found that after 1 year there was a 46.3% sustained clinical improvement rate in individuals with treated TASC II type D lesions, which was a significant improvement from baseline, with a 97.3% limb salvage rate in CLI patients (Davaine et al., 2012). These are promising results – stenting can be effective in long lesions; however, it is currently not the clinical gold standard. We believe that the biomimetic created here would be useful for primary studies of stents, prior to clinical trials – by using the biomimetic in research laboratories, it would allow for multiple tests of initial vessel patency and stent delivery to be conducted. Given the relative simplicity of this method of biomimetic creation, as many as required could be produced – this is in contrast with testing in cadaveric material, which is more difficult to source and may not be suitable for multiple uses.

Furthermore, there remains some uncertainty about which method of balloon angioplasty is most suitable for TASC II type D lesions such as CTOs. There are two types of balloon angioplasty typically used for CTO interventions – the aforementioned drug‐coated balloon angioplasty or cutting balloon angioplasty. Some would suggest that cutting balloons, which longitudinally score the vessel, are more effective than any others, as they better prepare the vessel prior to stenting and thus help to reduce restenosis rates (Lee et al., 2002). However, some studies have shown that cutting balloons are not effective for TASC II type D lesions (Horie et al., 2020). Therefore, by creating a biomimetic with calcium deposits printed in a ceramic material, which more closely resembles the mechanical properties of true CTO calcification, there is potential to further investigate the interactions of current cutting balloons with severe calcification and improve their design so that they may be utilised for clinical treatment of such lesions in future.

Indeed, printing the calcium deposits in a bone replacement material such as Lithoz LithaBone TCP 300 (a tricalcium phosphate‐based ceramic) or Lithoz LithaBone HA 400 (a hydroxyapatite material) (https://www.lithoz.com/en/our‐products/materials, Lithoz GmbH, Austria) would also be useful for studying the effect of devices which use energy to cross CTOs, such as medical lasers, mechanical vibration catheters and ultrasonic dissection catheters (Sakes et al., 2016). These materials would better mimic the bone‐like properties of calcium deposits and thus would prove useful in innovating these devices, as it would allow the fragmentation of large deposits to be studied.

## 5 CONCLUSIONS

Here we have described a replicable workflow for further characterising the complex anatomy of vascular CTOs and creating useful 3D‐printed models of diseased arteries, which may prove useful for medical device testing. While the samples available for this study were not true CTO samples, they enabled us to investigate the structure and location of calcium deposits, a major CTO component which is notoriously difficult to cross with conventional guidewires. Furthermore, the effectiveness of a previously described high‐resolution μCT technique for imaging whole arteries was demonstrated. The technique provides detail on both the calcified and soft tissue components of an arterial lesion, with the resulting segmentation process important for developing CTO biomimetics with multi‐material detail. This may enable further study of CTO anatomy and development of effective interventional tools to improve patient outcomes.

## CONFLICT OF INTEREST

The authors report no conflicts of interest.

## AUTHOR CONTRIBUTIONS

Conception and Design: Garry P. Duffy, Peter Dockery, Muireann O'Reilly. Analysis and Interpretation: Muireann O'Reilly. Data Collection: Muireann O'Reilly, Rachel Beatty, Shauna McBride, Benjamin Brennan. Writing the Article: Muireann O'Reilly. Critical Revision of Article: Garry P. Duffy. Final Approval of Article: Muireann O'Reilly and Garry P. Duffy. Statistical Analysis: Not applicable. Obtained Funding: Peter Dockery. Overall Responsibility: Garry P. Duffy.

## Supporting information


Video S1

## Data Availability

Data sharing is not applicable to this article as no new data were created or analyzed in this study.
